# Faecal miRNA profiles associated with age, sex, BMI, and lifestyle habits in healthy individuals

**DOI:** 10.1038/s41598-021-00014-1

**Published:** 2021-10-19

**Authors:** Antonio Francavilla, Amedeo Gagliardi, Giulia Piaggeschi, Sonia Tarallo, Francesca Cordero, Ruggero G. Pensa, Alessia Impeduglia, Gian Paolo Caviglia, Davide Giuseppe Ribaldone, Gaetano Gallo, Sara Grioni, Giulio Ferrero, Barbara Pardini, Alessio Naccarati

**Affiliations:** 1 Italian Institute for Genomic Medicine (IIGM), c/o IRCCS Candiolo, Candiolo, Turin, Italy; 2 Candiolo Cancer Institute, FPO-IRCCS, Candiolo, Turin, Italy; 3 Department of Computer Science, University of Turin, Turin, Italy; 4 Division of Gastroenterology, Department of Medical Sciences, University of Turin, Turin, Italy; 5 Department of Medical and Surgical Sciences, University of Catanzaro, Catanzaro, Italy; 6 Epidemiology and Prevention Unit, Fondazione IRCCS Istituto Nazionale Dei Tumori Di Milano, Milan, Italy; 7 Department of Clinical and Biological Sciences, University of Turin, Turin, Italy

**Keywords:** Risk factors, miRNAs, Next-generation sequencing

## Abstract

For their stability and detectability faecal microRNAs represent promising molecules with potential clinical interest as non-invasive diagnostic and prognostic biomarkers. However, there is no evidence on how stool miRNA profiles change according to an individual’s age, sex, and body mass index (BMI) or how lifestyle habits influence the expression levels of these molecules. We explored the relationship between the stool miRNA levels and common traits (sex, age, BMI, and menopausal status) or lifestyle habits (physical activity, smoking status, coffee, and alcohol consumption) as derived by a self-reported questionnaire, using small RNA-sequencing data of samples from 335 healthy subjects. We detected 151 differentially expressed miRNAs associated with one variable and 52 associated with at least two. Differences in miR-638 levels were associated with age, sex, BMI, and smoking status. The highest number of differentially expressed miRNAs was associated with BMI (n = 92) and smoking status (n = 84), with several miRNAs shared between them. Functional enrichment analyses revealed the involvement of the miRNA target genes in pathways coherent with the analysed variables. Our findings suggest that miRNA profiles in stool may reflect common traits and lifestyle habits and should be considered in relation to disease and association studies based on faecal miRNA expression.

## Introduction

In the last years, an increasing interest in understanding how individuals’ behaviour can affect human health has been witnessed. Environmental factors such as diet, physical activity, smoking habits, and alcohol consumption, in addition to individual traits, including sex, age, and Body Mass Index (BMI), have been established as risk factors for many diseases, including neurodegenerative and metabolic disorders, as well as cancer^1^. However, the molecular mechanisms behind these relationships have not been exhaustively clarified^2^. Epigenetic modifications, such as DNA methylation, histone acetylation, nucleosome positioning, and alteration of non-coding RNA profiles are some of the modalities whereby the environment affects gene expression and ultimately impacts individuals’ health^3^. Among the wide range of non-coding RNA molecules, small non-coding RNAs (sncRNAs) represent a promising subclass of great interest for their potential clinical applications in the diagnosis and therapies of several diseases^4^. microRNAs (miRNAs), the most studied sncRNAs, post-transcriptionally regulate many messenger RNAs with the primary function of fine modulation of gene expression, thus affecting many physiological and developmental processes^5^. These molecules are stable and easily detectable in various human biospecimens, including biofluids and, combined with the fact that they are dysregulated in several diseases, they represent ideal candidates as non-invasive biomarkers^6, 7^. These aspects explain the impressive research on miRNAs over the last years in biomedicine.

In contrast, the relationships between miRNAs and individual traits/lifestyle factors have not been explored at the same pace. So far, most of the studies have evaluated miRNA expression levels in relation to age, sex, and BMI almost exclusively in whole-blood, plasma, or serum^8–12^. The influence of smoking on miRNA expression has also been frequently studied. Indeed, miRNAs resulted dysregulated in plasma, peripheral blood cells, and human airway epithelium of both current and former smokers compared to non-smokers healthy subjects^13–15^. On the other hand, the effect of physical activity on circulating miRNA levels has been mainly investigated in intervention studies^16, 17^. Very little is known regarding the impact of other important lifestyle habits such as alcohol or coffee consumption on miRNA expression levels in healthy subjects. To the best of our knowledge, only one study investigated the effects of alcohol intake on miRNA expression levels in healthy volunteers^18^ while no study focused on coffee intake, although this beverage is largely consumed worldwide and is widely studied in relation to cancer and other diseases^19–21^. Apart from a few exceptions^17^, all the available studies presented some limitations, such as the small number of subjects investigated and/or a limited number of miRNAs analysed (mainly evaluated by arrays and/or RT-qPCR).

Among the biospecimens where miRNAs can be detected, stool are emerging as a relevant surrogate tissue for the non-invasive diagnosis of gastrointestinal disorders^22–24^. Exploring faecal miRNAs has several positive aspects: miRNAs from exfoliated colonocytes are directly and continuously released into the intestinal lumen being easily detectable in faecal samples with a minimal invasiveness^25^. Importantly, recent discoveries highlighted an interplay between faecal miRNAs and human microbiota, whose composition varies in relation to different lifestyles and gastrointestinal conditions^22, 23, 26^. In this respect, clarifying the relationship between stool miRNA expression levels and common traits and lifestyle habits may be essential^27^.

In the present study, we investigated miRNA expression levels by small RNA sequencing (sRNA-seq) in stool of 335 healthy volunteers in relation to sex, age, BMI, smoking, alcohol and coffee consumption, and physical activity to understand their possible modulatory effects on the human faecal miRNome. We found several miRNAs associated with one or more of the investigated variables, with the largest number of differentially expressed miRNAs (DEmiRNAs) in relation to BMI, smoking, and coffee consumption. Overall, our findings revealed considerable relationships which should be considered in stool miRNA-based biomarkers research studies in the context of personalised medicine.

## Results

### Study population

**Figure 1:**
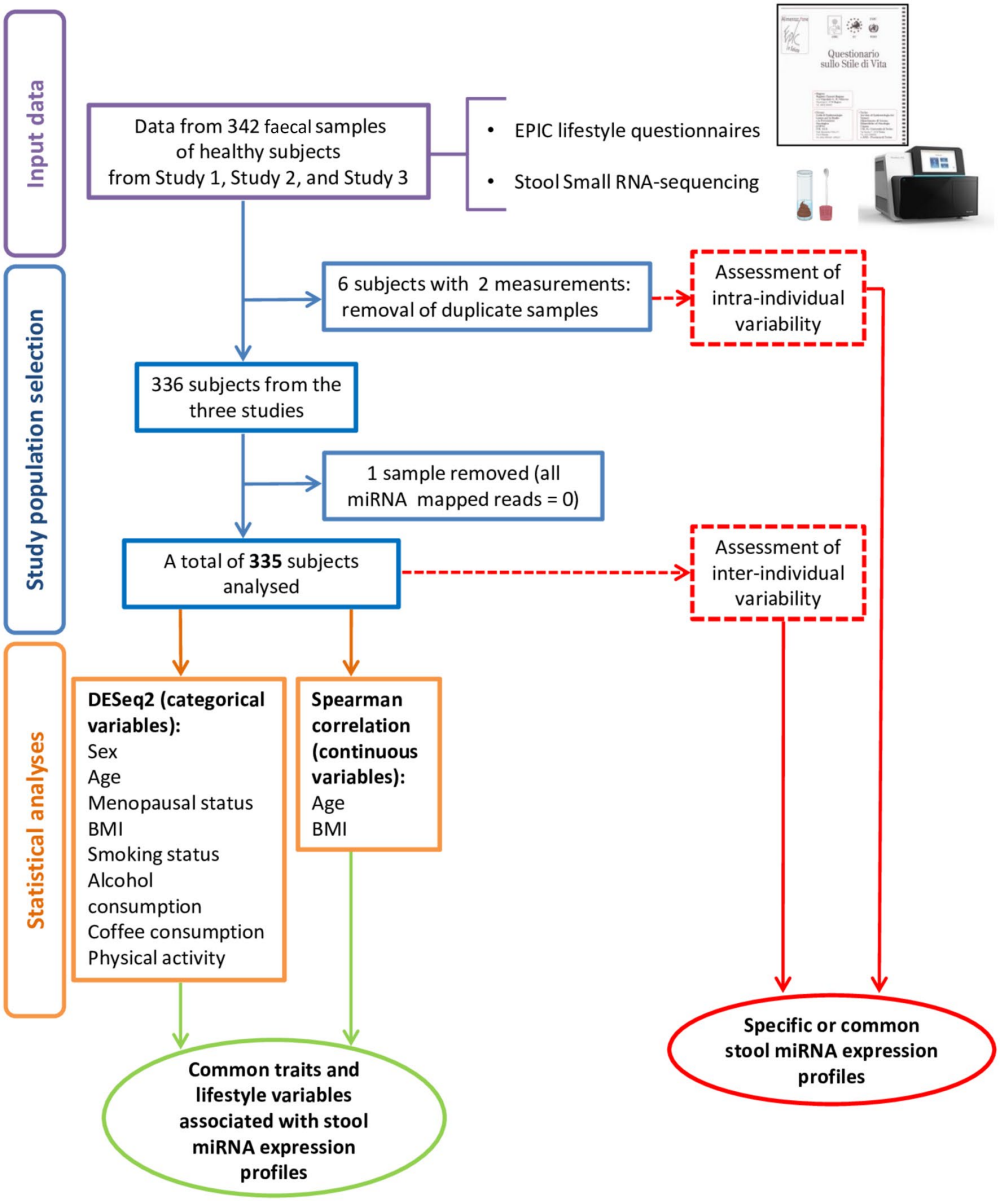
Workflow of the study.

A total of 342 faecal samples were collected from healthy subjects recruited in different studies. Six subjects provided a second stool sample one year after the first collection: for them, only data from the first sampling were considered in the analyses while the second sample was used to assess intra-individual variability. One subject was excluded because few sRNA-Seq reads were aligned on miRNA sequences. The final study population consisted of 335 subjects provided by both stool sRNA-seq data and lifestyle questionnaires (Fig. 1). Subjects had an average age of 44.7 ± 14.7 years old (range: 18–81) and 63.6% were females (Table 1).

**Table 1:** Study population characteristics.

	Female (N = 213)	Male (N = 122)	Overall (N = 335)
**Age years**			
Mean ± SD	43.9 ± 14.5	46.1 ± 15.0	44.7 ± 14.7
Median [Min, Max]	44.0 [18.0, 80.0]	48.0 [19.0, 81.0]	45.0 [18.0, 81.0]
**Age tertiles (%)**			
18–37	84 (39.4%)	38 (31.1%)	122 (36.4%)
37–53	66 (31.0%)	45 (36.9%)	111 (33.1%)
53–81	63 (29.6%)	39 (32.0%)	102 (30.5%)
BMI*			
Mean ± SD	22.4 ± 3.7	24.5 ± 3.6	23.1 ± 3.8
Median [Min, Max]	21.9 [15.4, 39.9]	24.1 [17.2, 35.8]	22.6 [15.4, 39.9]
Missing (%)	5 (2.3%)	6 (4.9%)	11 (3.3%)
BMI Classes (%)*			
Underweight	23 (10.8%)	2 (1.6%)	25 (7.5%)
Normal	145 (68.1%)	70 (57.4%)	215 (64.2%)
Overweight	32 (15.0%)	34 (27.9%)	66 (19.7%)
Obese	8 (3.8%)	10 (8.2%)	18 (5.4%)
Missing	5 (2.3%)	6 (4.9%)	11 (3.2%)
**Smoking status (%)**			
Current smoker	37 (17.4%)	20 (16.4%)	57 (17.0%)
< 16 cigs/day	28 (13.1%)	13 (10.6%)	41 (12.2%)
> 16 cigs/day	9 (4.2%)	7 (5.7%)	16 (4.8%)
Former smoker	50 (23.5%)	44 (36.1%)	94 (28.1%)
Never smoker	125 (58.7%)	56 (45.9%)	181 (54.0%)
Missing	1 (0.4%)	2 (1.6%)	3 (0.9%)
Alcohol consumption (%)**			
Non drinkers	23 (10.8%)	6 (4.9%)	29 (8.7%)
Low intake drinkers	151 (70.9%)	79 (64.8%)	230 (68.7%)
High intake drinkers	38 (17.8%)	37 (30.3%)	75 (22.4%)
Coffee consumption (%)***			
Non drinkers	89 (41.8%)	46 (37.7%)	135 (40.3%)
Low intake drinkers	96 (45.1%)	53 (43.4%)	149 (44.5%)
High intake drinkers	27 (12.7%)	23 (18.9%)	50 (14.9%)
**Physical activity (%)**			
Active	14 (6.6%)	7 (5.7%)	21 (6.3%)
Moderately active	63 (29.6%)	45 (36.9%)	108 (32.2%)
Moderately inactive	72 (33.8%)	42 (34.4%)	114 (34.0%)
Inactive	63 (29.6%)	27 (22.1%)	90 (26.9%)
Missing	1 (0.4%)	1 (0.9%)	2 (0.6%)
**Menopausal status (%)**			
Premenopausal	132 (62.0%)	-	-
Postmenopausal	77 (36.2%)	-	-
Missing	4 (1.8%)	-	-

*BMI was categorized according to the World Health Organization guidelines as underweight (< 18.5 kg/m^2^), normal weight (18.5–24.9 kg/m^2^), overweight (25.0–29.9 kg/m^2^) and obese (30.0–34.9 kg/m^2^).

**Alcohol consumption was categorized according to the alcohol intake measured in gr/day: non-drinkers (0 gr/day), low intake drinkers (< 24 gr/day for males and < 12 gr/day for females) and high intake drinkers (> 24 gr/day for males and > 12 gr/day for females).

***Coffee consumption was categorized according to the coffee intake measured in g/day: non-drinkers (0 gr/day), low intake drinkers (1–16 gr/day) and high intake drinkers (> 16 gr/day).

### Stool miRNA profiles and analysis of the intra/inter-individual expression variability

On average, 10.3 million single-end reads per sample were obtained from sRNA-Seq with a median of 64,281 reads (0.92%) assigned to human miRNA annotations (Supplementary Table 1A). In total, 3,277 miRNAs (93% of our miRNA reference) were detected in at least one subject, but an extensive variability was observed among individual miRNA profiles (ranging from 188 to 1,949 detected miRNAs per sample), with a median of 594 miRNAs detected in stool of the studied population. The chromosomal distribution of all the stool miRNAs detected was compared with that of the total reference miRNome investigated (n = 3,524): an average of 93.7% of detected miRNAs per chromosome was observed, with the highest percentage on chromosome Y and the lowest on chromosome 2 (100% and 88.2% of the total miRNA coding genes, respectively; Supplementary Table 1B).

Four hundred and forty-nine miRNAs (13.8%) were detected in at least half of the analysed samples (Supplementary Table 1B). Among them, nine miRNAs (*miR-320e-5p,* miR-607*, miR-647-3p, miR-1246-3p,* miR-1302*,* miR-3125, miR-5698, miR-6075, and miR-6777-5p) were detected in all the samples analysed. miR-3125 was characterised by the highest median expression levels (2,051 reads), followed by miR-6075 (921 reads), and *miR-1246-3p* (884 reads).

**Figure 2:**
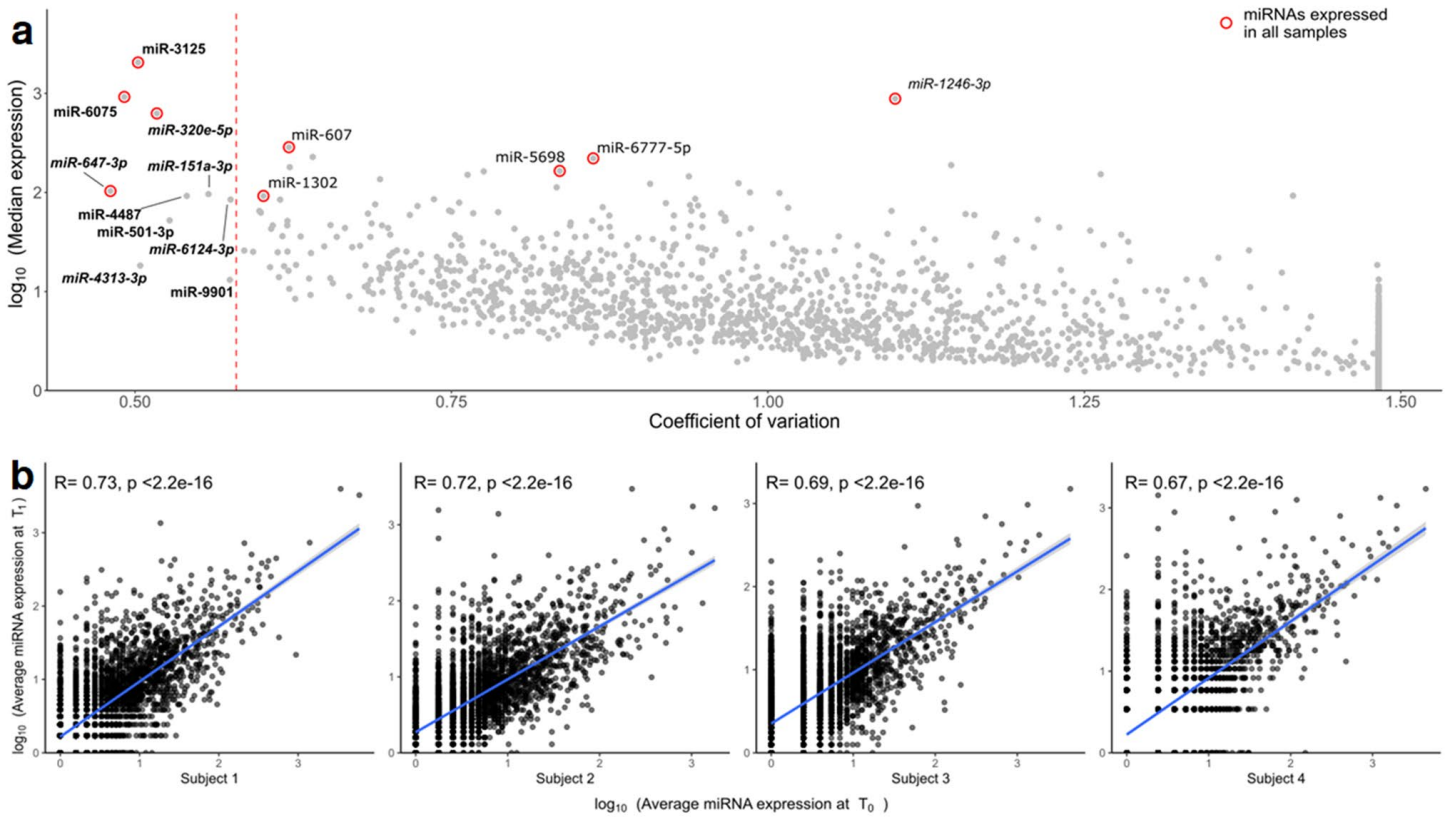
(**a**) Scatter plot showing the stool miRNA inter-individual variability expressed as a relation between median expression levels and coefficient of variation (CV). Red dots represent miRNAs detected in all samples. On the left of the dotted line are reported those miRNAs characterised by the lowest CV. (**b**) Scatter plots showing the stool miRNA intra-individual variability of four selected subjects who provided a stool sample at two different time points.

To evaluate the inter-individual variability of stool miRNA expression levels across the study population, a coefficient of variation (CV) was calculated for all miRNAs with a median read expression level of at least one (n = 2,031). The resulting coefficients were used to rank miRNAs according to their expression variability (Supplementary Table 1C). *miR-647-3p,* miR-6075, and miR-3125, also expressed in all samples, showed the lowest expression variability (CV ranging from 0.48 to 0.50, Fig. 2a).

Repeated samples collected from six subjects were used to assess the stability of stool miRNA expression levels over time. A second faecal sample was collected approximately one year after the first collection (min = 378 days, max = 560 days). No significant changes in lifestyle habits were reported from the questionnaires compiled by the participants on both occasions. A Spearman correlation analysis between miRNA expression levels at the two time points showed a correlation coefficient ranging from 0.54 (p < 0.001) to 0.72 (p < 0.0001) (Fig. 2b). A Wilcoxon paired test performed on the set of nine miRNAs detected in all samples showed that they were also expressed in the repeated samples and did not show any significant difference in mean expression levels (p > 0.05), except for miR-3125 (Supplementary Table 1D).

### miRNA profiles in relation to common traits

**Figure 3:**
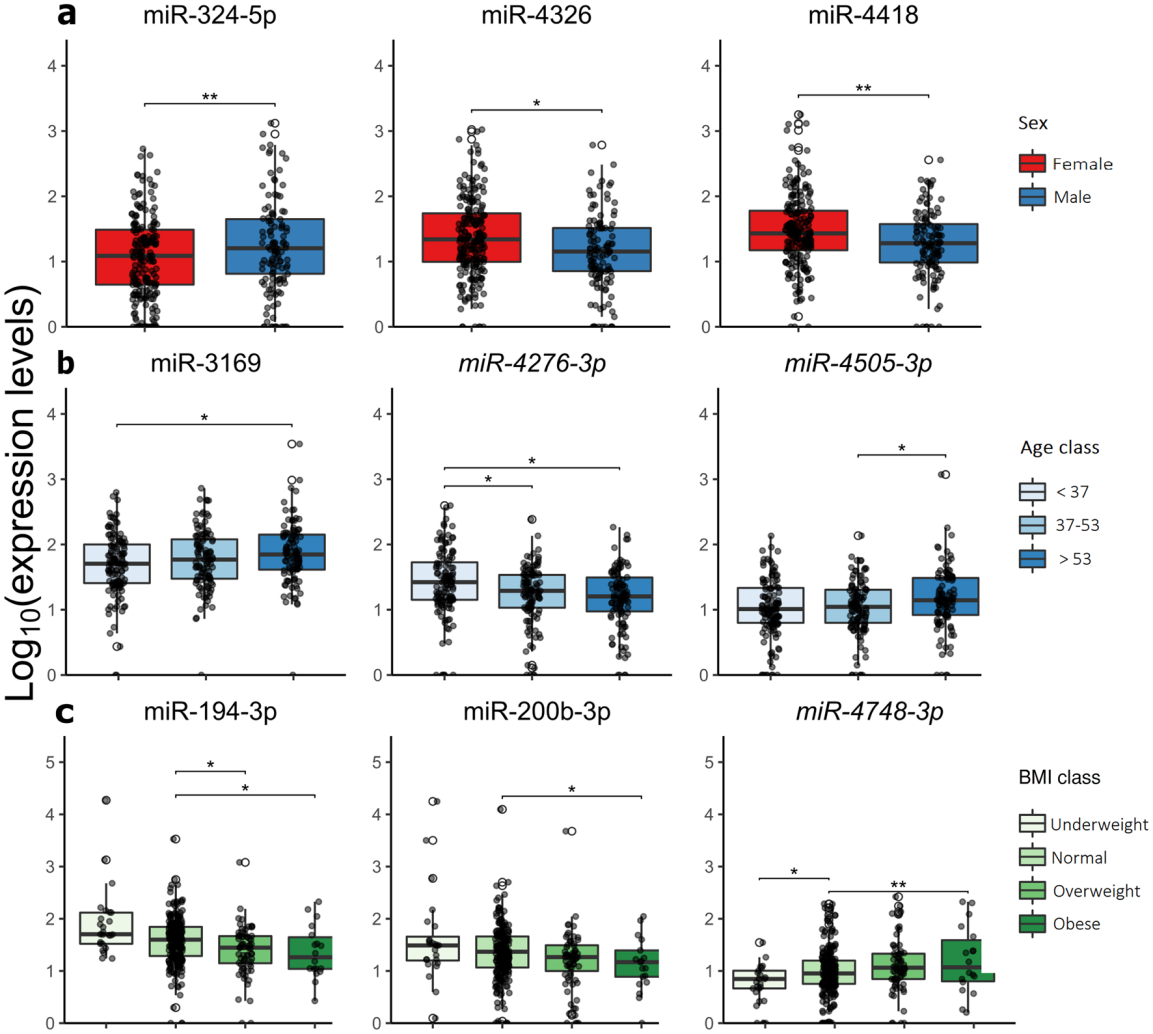
(**a–c**) Box plots showing the expression levels of selected differentially expressed miRNAs among individuals stratified according to the investigated common trait: sex (**a**), age (**b**), and BMI (**c**). P-values were computed by DESeq2 and adjusted using the FDR method. ***adj.p < 0.001, **adj.p < 0.01, *adj.p < 0.05.

miRNA expression levels were explored in relation to sex, age, menopausal status, and BMI. Initially, miRNA profiles of 122 males and 213 females were compared and nine DEmiRNAs were observed between sexes (Supplementary Table 2). Specifically, five were up-regulated (miR-324-3p, miR-324-5p, miR-1255b-5p, miR-3935, and miR-4675) and four down-regulated (*miR-3615-5p*, miR-4326, miR-4418, and miR-4632-5p) in males. The expression levels of miR-324-5p, miR-4326, and miR-4418 are reported as an example of DEmiRNAs associated with sex (Fig. 3a).

Considering the age distribution of the study population, miRNA profiles were investigated comparing three categories: < 37 (n = 122), 37–53 (n = 111), and > 53 (n = 102) years old. In total, 19 DEmiRNAs were identified in at least one comparison among these categories (Supplementary Table 2). miR-1231 and *miR-4276-3p* were down-regulated and miR-4487 was up-regulated in subjects of the age class 37–53 compared with those of the age class < 37. Comparing subjects of age class > 53 with those of the class < 37, 7 DEmiRNAs (6 down- and miR-3169 up-regulated in the older group) were identified. Between the age classes 37–53 and > 53, 10 DEmiRNAs (3 down- and 7 up-regulated in the eldest group) were identified. Progressive reduced expression levels with increasing age were observed across categories for *miR-4276-3p* and increased for miR-3169 and *miR-4505-3p* (Fig. 3b).

For the 19 DEmiRNAs, a similar expression pattern among age classes was observed when the analysis was performed considering males and females separately (Supplementary Table 2). However, only miR-550a-3-5p was significantly down-regulated in males of the age class 37–53 with respect to the other two classes. Additionally, 13 and 6 DEmiRNAs were specifically associated with age in males and females, respectively (Supplementary Table 2).

The relationship between stool miRNA expression levels and age was also explored by Spearman correlation analysis, considering the whole study population or stratified by sex. In the whole population, four miRNAs (miR-922, miR-3619-3p, *miR-4276-3p*, and miR-8074) decreased with age (-0.26 < SCC < -0.19; adj. p < 0.05). In females, the expression of miR-8074 was inversely correlated with age (SCC = -0.28, adj. p < 0.001) while in males no significant correlation was found (Supplementary Table 3).

Stool miRNA expression levels of women in premenopausal status (n = 132) were compared with those in post menopause (n = 77). Only *miR-3615-5p* was significantly down-regulated in postmenopausal women (Supplementary Table 2). This miRNA was also significantly more expressed in females compared to men.

miRNA levels in relation to BMI were assessed comparing obese (n = 18), overweight (n = 66), and underweight (n = 25) individuals with normal weight subjects (n = 215). A total of 92 miRNAs were significantly differentially expressed in at least one comparison (Supplementary Table 2). In the obese group, ten and eight miRNAs were respectively up- and down-regulated, 69 miRNAs (64 down- and five up-regulated) were altered in the overweight group, and, finally, in the underweight group, 18 DEmiRNAs (13 down- and 5 up-regulated) were identified. Many of the DEmiRNAs showed a trend of expression going from underweight to obese, including miR-194-3p, miR-200b-3p, and *miR-4748-3p* (Fig. 3c and Supplementary Table 2).

Stratifying for sex, the 92 above mentioned DEmiRNAs showed a coherent trend of expression either in males or in females (Supplementary Table 2) with 4 and 18 DEmiRNAs also significant in males and females, respectively. Additional differentially expressed miRNAs were found in males (n = 4) or females (n = 33) only.

The stool miRNA expression profiles according to BMI (considered as a continuous variable) were further investigated by Spearman correlation analysis. With an increasing BMI, the expression levels of 22 miRNAs significantly decreased (-0.16 < SCC < -0.24, adj. p < 0.05) while 4 miRNAs increased (-0.22 < SCC < 0.17, adj. p < 0.05) (Supplementary Table 3). Twenty out of the 26 miRNAs significantly correlated with BMI were also differentially expressed among the BMI groups. No significant correlations were observed after the stratification for sex.

### miRNA profiles according to lifestyle habits

miRNA levels were further investigated in relation to smoking status, alcohol, and coffee consumption as well as physical activity.

**Figure 4:**
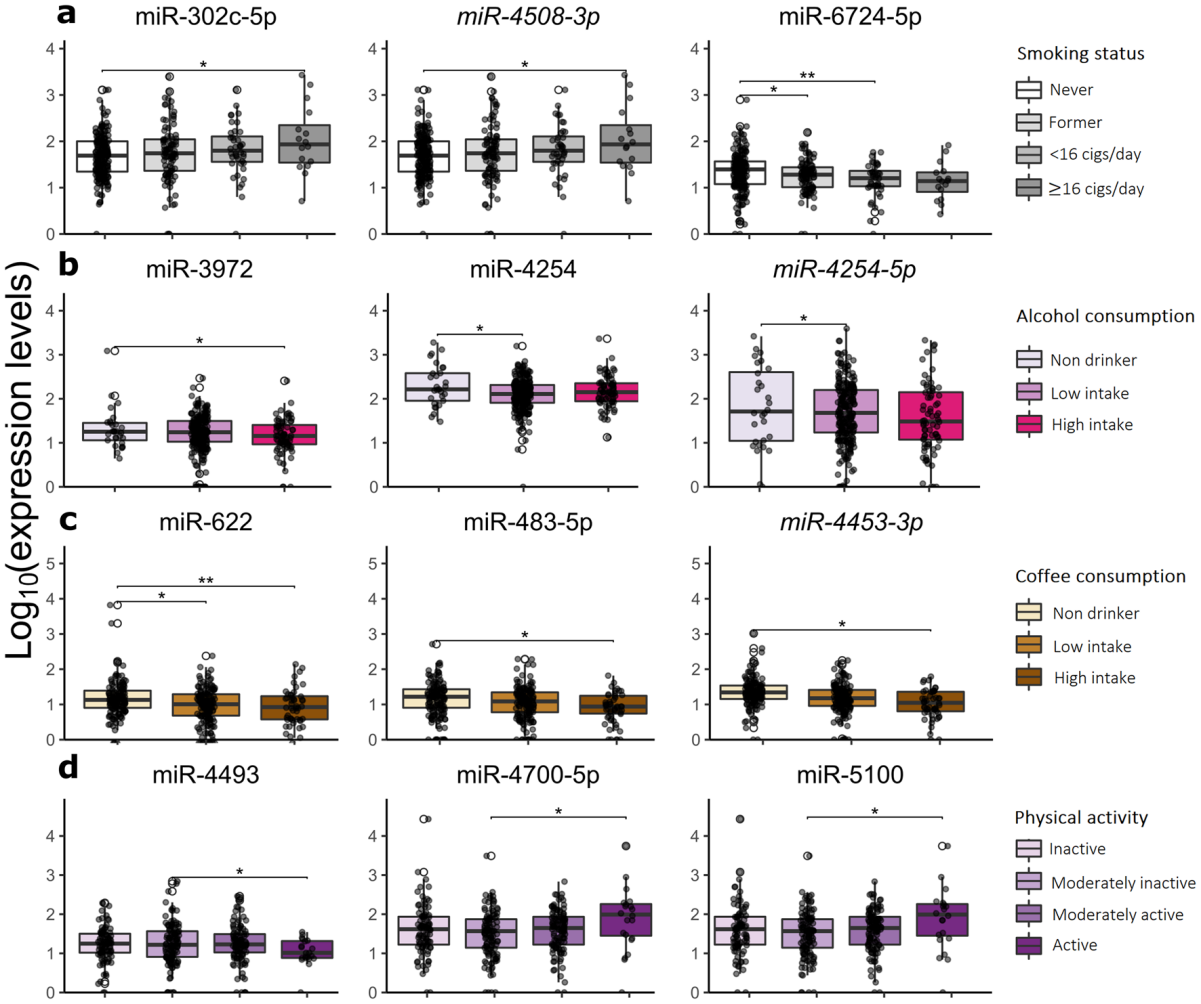
(**a–d**) Box plots showing the expression levels of selected DEmiRNAs among individuals stratified according to the investigated lifestyle habits: smoking status (**a**), alcohol (**b**) or coffee (**c**) consumption, and physical activity (**d**). P-values were computed by DESeq2 and adjusted using the FDR method. ***adj.p < 0.001, **adj.p < 0.01, *adj.p < 0.05.

For smoking habits, miRNA levels were analysed comparing subjects who smoked more than 16 cigs/day (n = 16), those smoking less than 16 cigs/day (n = 41) and former smokers (n = 94) with never smokers (n = 181). Overall, 84 DEmiRNAs were identified from the three comparisons (Supplementary Table 2). Comparing individuals who smoke more than 16 cigs/day with non-smokers, 59 DEmiRNAs were identified (50 up- and nine down-regulated) while 22 miRNAs were differentially expressed in those who smoke less than 16 cigs/day compared to non-smokers (three up- and 19 down-regulated). Interestingly, *mir-8075-5p* and miR-12128 were down-regulated in both smoking categories compared to non-smokers. Finally, 13 miRNAs were differentially expressed in former smokers vs non-smokers, with *miR-5090-3p* up-regulated and the other 12 DEmiRNAs down-regulated in the former group. Some of the 84 DEmiRNAs associated with smoking showed a trend of expression from non-smoker individuals to those who smoke more than 16 cigs/day (Supplementary Table 2). miR-302c-5p, miR-4508-3p and miR-6745-5p are reported in Fig. 4a as examples of such trends.

A similar expression pattern was observed for the 84 DEmiRNAs when the population was stratified by sex, with 14 and 26 out of 84 DEmiRNAs significantly dysregulated in men and women, respectively. Other additional sex-specific DEmiRNAs were uniquely identified in men (n = 17) and women (n = 14) (Supplementary Table 2).

The relationship between miRNA expression levels and alcohol consumption was assessed comparing non-drinkers (n = 29) with low (n = 230) and high (n = 75) intake drinkers. Comparing high intake drinkers with non-drinkers, miR-3972 was down-regulated in the latter, whereas in low intake drinkers vs non-drinkers, miR-4254 and *miR-4254-5p* were down-regulated, and miR-6895-3p up-regulated in drinkers. The expression levels of miR-3972, miR-4254, and *miR-4254-5p,* characterised by a trend of expression in non-drinkers, low, and high intake drinkers, are reported in Fig. 4b. The aforementioned 4 alcohol-related DEmiRNAs showed a similar trend of expression also stratifying by sex, with miR-4254 and miR-3972 being significant in males and *miR-4254-5p* and miR-6895-3p in females. Moreover, other eight DEmiRNAs were uniquely observed in males and 11 in females in both drinking categories vs non-drinkers.

miRNA profiles in relation to coffee consumption were studied comparing low (n = 149) and high (n = 50) intake coffee drinkers with non-drinkers (n = 135). In high intake coffee consumers vs non-drinkers, 44 miRNAs were differentially expressed, with three up- and 41 down-regulated, in drinkers (Supplementary Table 2). Five out of these 44 miRNAs were also differentially expressed with the same expression trend in low coffee drinkers compared to non-drinkers: miR-1281 was up-regulated and miR-622, miR-1231, miR-7515-5p, and miR-8053 were down-regulated in drinkers. Three miRNAs characterised by decreasing expression levels going from non-drinkers to high drinker subjects are reported in Fig. 4c.

Performing the same comparisons and stratifying the population according to sex, similar trends of expression were observed for the 44 DEmiRNAs previously reported. Two additional miRNAs in males and seven in females were uniquely identified (Supplementary Table 2).

The stratification of the study population according to the physical activity identified the following categories: inactive (n = 90), moderately inactive (n = 114), moderately active (n = 108), and active (n = 21) subjects. Comparing each of the first three categories against active individuals, 11 DEmiRNAs were identified (Supplementary Table 2). Six miRNAs, five down- and one up-regulated (miR-182-3p), resulted from the comparison between inactive vs active subjects. Also six DEmiRNAs (two up- and four down-regulated) were found in moderately inactive individuals and, finally, seven miRNAs (four up- and three down-regulated) in those moderately active. Two DEmiRNAs (miR-4493 and miR-4700-5p) with significant increasing expression levels in the active category and miR-646 with a mild trend of up-regulation going from inactive to the active group are reported in Fig. 4d.

The same comparisons were also performed stratifying according to sex, confirming similar trends of expression for the 11 DEmiRNAs in both males and females. However, among them, miR-646, miR-5090-3p, and miR-12121 were found significantly altered in females and just miR-4700-5p altered in males. Additionally, eight DEmiRNAs were found only in males (n = 4) or females (n = 4) (Supplementary Table 2).

### Overview of common miRNAs altered among investigated variables

**Figure 5:**
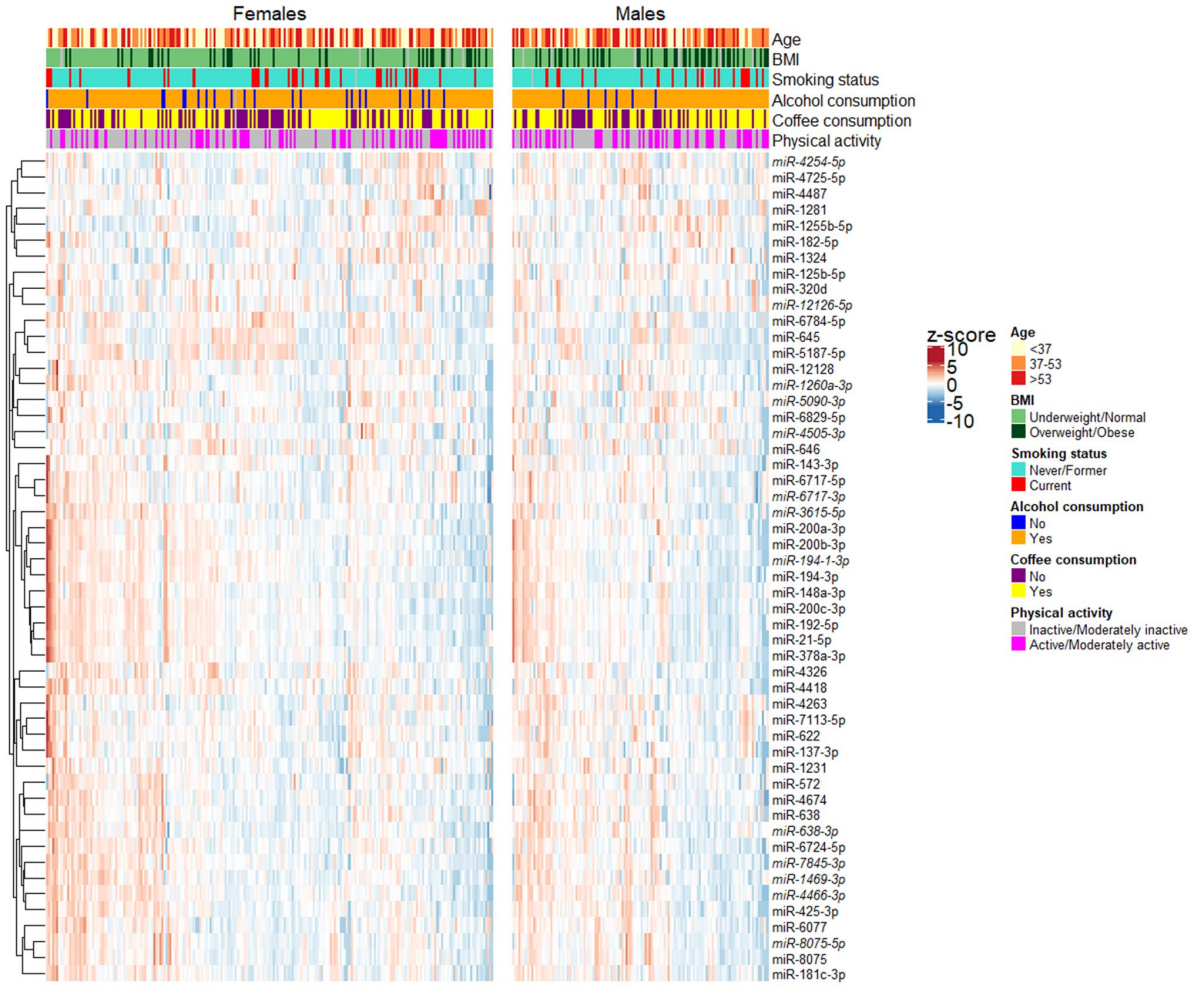
Heatmap reporting, separately for males and females, the results of the hierarchical clustering of 52 DEmiRNAs significantly associated with two or more variables. For each DEmiRNA, the z-score of log10 read count for each sample is reported.

From a total of 3,041 miRNAs detected, 151 (5%) were associated with at least one of the analysed common traits or lifestyle habits while 52 DEmiRNAs were significant in two or more comparisons (Supplementary Table 2). Considering separately for males and females the stool levels of the latter group of DEmiRNAs, a subtle clustering of miRNAs emerged for both sexes, mainly related to smoking habit, BMI and coffee consumption, as reported in the heatmap in Fig. 5.

To test the temporal stability of all the identified stool DEmiRNAs expression levels in repeated samples collected from the same individuals, a Wilcoxon paired test was performed between the available samples collected at two time points. One hundred and seventy-three miRNAs (85.2%) showed no significant variation between the two measurements (p ≥ 0.05, Supplementary Table 1D). The remaining 30 DEmiRNAs (among which two variables shared 11 miRNAs), showing a significant variability between the two time points, were mostly related to BMI (n = 13), smoking habit (n = 14) or coffee consumption (n = 11).

### Target gene enrichment analysis

Functional enrichment analysis was performed to explore whether independent sets of faecal DEmiRNAs identified in this study were involved in relevant biological processes for the investigated variables. The analysis, carried out considering the validated target genes of DEmiRNAs, revealed a total of 298 significantly enriched terms (Supplementary Table 4). Two hundred and eleven, six and 81 terms were enriched for GO, KEGG, and REACTOME gene set libraries, respectively. An overlap of five REACTOME enriched terms among different variables was observed and reported in Supplementary Table 4.

## Discussion

**Figure 6:**
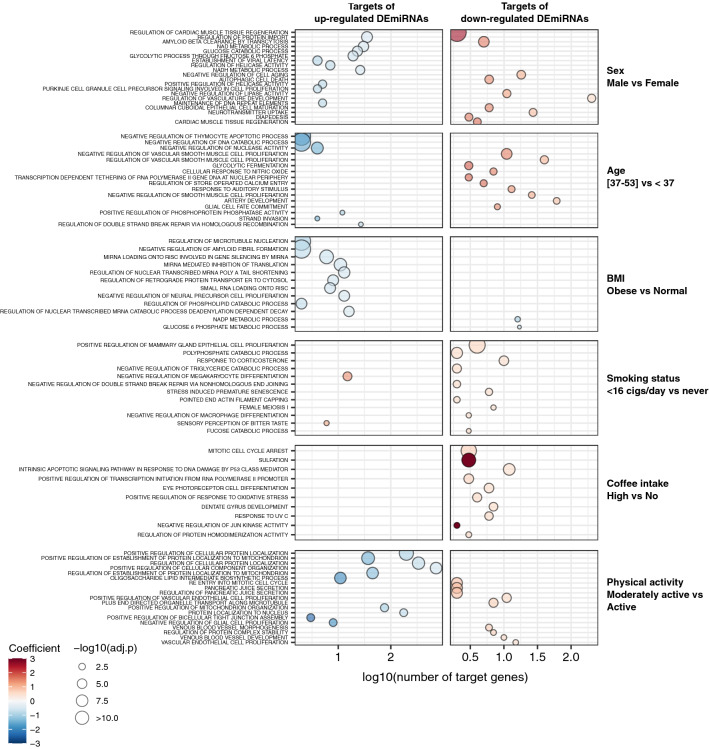
Dot plots showing the statistical significance of the GO Biological Process enriched terms considering the DEmiRNA validated target genes obtained for each of the analysed variables. The size of the dots is proportional to the significance of the enrichment while the number of target genes belonging to each term is reported on the x-axis. The colour code refers to the coefficient computed by RBiomirGS. Negative (in blue) and positive (in red) coefficients represent processes predicted to be down-regulated or up-regulated, respectively, based on the different miRNA expression levels between comparisons.

An increasing number of observations has highlighted the clinical and nutritional potentialities of stool miRNAs^22, 25, 28^. However, there is still limited evidence on their expression levels in relation to common traits and lifestyle habits in healthy individuals. Our study aimed at filling this gap, providing the first evidence on how the faecal miRNome expression is affected by a set of common variables investigated in a population of 335 healthy subjects. A total of 203 miRNAs were significantly associated with at least one of the considered variables, with 52 associated with more than one. Several miRNAs showed a differential expression in agreement with other studies on blood or tissue samples ^10, 29, 30^. For example, differences in miRNA expression levels related to sex have been previously reported ^27, 29, 31^. Interestingly, miR-324-5p, up-regulated in males in our study, was also reported up-regulated in healthy brain tissue of males compared to females^29^. Enrichment analyses of the validated targets of the sex-associated DEmiRNAs showed several enriched terms (Fig. 6 and Supplementary Table 4), including metabolic terms such as *glucose catabolic process, glycolytic process through fructose 6 phosphate*, *NR1H2 NR1H3 regulate gene expression to control bile acid homeostasis*, and others linked to the senescence (*cell aging*, *cellular senescence)* which have already been observed differently regulated according to sex^32, 33^.

Alterations in miRNA expression levels associated with aging have also been reported in various biospecimens^8, 34^. In stool samples, we observed a group of miRNAs affected by aging, with the majority of them down-regulated in older subjects. Interestingly, among these miRNAs miR-320d, miR-638, miR-922, miR-1229-5p, and miR-1231 were found strongly decreased with age in whole blood samples from 1,334 healthy individuals by Fehlmann et al.^10^. Enrichment analysis based on the age-related miRNAs revealed their involvement in *cellular sphingolipid homeostasis*, *CREB phosphorylation*, *negative regulation of vascular smooth muscle cell proliferation*, *cellular response to reactive nitrogen species* and *regulation of store operated calcium entry,* which have all been largely described modulated by aging^35–38^ (Fig. 6).

To the best of our knowledge, the relationship between menopausal status and miRNA expression levels has been investigated only in relation to menstrual cycle disorders or hormones regulation and not in healthy women^39–41^. We reported, for the first time, one miRNA, *miR-3615-5p*, down-regulated in faecal samples of postmenopausal women. Notably, this miRNA resulted also significantly up-regulated in females compared to males and down-regulated with increasing BMI. However, this observation should be carefully considered since miR-3615 overlaps the 5’UTR of *SLC9A3R1* gene and Kim et al. previously demonstrated that miR-3615 is among three miRNAs having a non-canonical biogenesis, i.e., from 5′ capped pre-miRNAs not requiring DROSHA for their maturation process^42^. Further experiments are needed to clarify the specificity of the observed expression of this miRNA in different biospecimens.

Compared to sex and age, we observed a higher number of faecal miRNAs associated with BMI. Many studies reported circulating miRNA dysregulation in relation to BMI, mainly focusing on overweight and obesity conditions in which most of the described DEmiRNAs were down-regulated. Such evidence highlighted the involvement of miRNAs in BMI-related pathways such as adipogenesis, insulin resistance and fatty acid and cholesterol metabolism^43^. In this study, 92 miRNAs were reported with variable expression levels according to different BMI. Among them, we found miR-143-3p with a decreasing expression as the BMI increased, as already described by Ameling et al. in plasma of healthy individuals^11^. This miRNA has a role in several cancers as tumor suppressor^44–46^; however, the literature available reported also its involvement in adipogenesis differentiation, obesity-related inflammation and insulin resistance, even if the trend of expression with BMI is not always concordant among the studies^47, 48^. Functional analysis of the target genes of the 92 DEmiRNAs associated with BMI highlighted enriched terms in pathways related to this variable (Fig. 6 and Supplementary Table 4). Among these, *glucose 6 phosphate metabolic process*, *NADP metabolic process* and *negative regulation of amyloid fibril formation,* which have been largely described in relation to BMI and obesity^49, 50^.

Overall, compared to the individual common traits, variables related to lifestyles showed a more substantial impact on faecal miRNA expression. Many DEmiRNAs resulted associated with smoking, including miR-21-5p, miR-148a-3p, miR-200b-3p, and miR-200c-3p, that were already found altered with similar trends in in vitro studies^51, 52^. The impact of smoking habit on miRNA expression levels has been reported on plasma, peripheral blood mononuclear cells, and small airway epithelium samples, showing a differential expression for several miRNAs in smokers compared to non-smokers^13–15, 30^. In agreement with the present work, a study on whole blood from 226 healthy individuals showed an up-regulation of miR-1229-5p and miR-4739 in current smokers compared to never^30^. Interestingly, an increased difference in miRNA expression levels was observed when smokers were stratified according to the number of cigarettes smoked. This finding suggests that the amount of smoking is probably more influential on miRNA levels detectable in stool than the smoking itself. Enrichment analysis of the DEmiRNA target genes associated with smoking identified many biological pathways (Fig. 6 and Supplementary Table 4), including *response to corticosterone,* involved in the immunosuppressive effect of nicotine^53^, *sensory perception of bitter taste* and *regulation of RUNX1 expression and activity* already described in relation to cigarette smoking^54, 55^.

The effect of alcohol consumption on miRNA expression has been primarily studied in liver disease and neuroinflammation^56–59^. However, the relationship between alcohol consumption and miRNA expression levels has been scarcely investigated in healthy individuals. One study explored miRNA expression levels before and after alcohol consumption in serum samples of healthy young individuals attending a social event. The authors observed that 265 miRNAs were up-regulated and miR-185-5p was down-regulated after alcohol consumption^18^. Analysing our data, we found four miRNAs significantly associated with alcohol consumption, but none of them overlapped with those of the previously described study. However, no significant miRNA target enriched terms were observed, as well as no other evidence for such miRNAs, highlighting the need for further research on this topic.

We also demonstrated a relationship between coffee intake and stool miRNA levels with 84 DEmiRNAs comparing high- and low-intake individuals against non-drinkers, with a prevalence of down-regulated miRNAs. Previous studies have reported the influence of coffee consumption on miRNA regulation^60^, though it is still unclear if these effects are due to caffeine itself or the combination with other coffee compounds^61, 62^. Target functional analyses showed several GO terms related to apoptosis and cell response to stress (Fig. 6 and Supplementary Table 4). This is in line with other studies that have investigated the relationship between caffeine and apoptosis^62, 63^.

Finally, 11 DEmiRNAs were found associated with a more active lifestyle. Although associations between physical activity and miRNA profiles have been already described, no study is available on stool miRNAs. Several studies, included in the review from Dufresne and colleagues^64^, have suggested that physical activity could influence miRNA expression levels in healthy individuals, especially those miRNAs that are muscle-related and regulated within muscle tissues (also called myomiRs)^65^. Functional analyses based on DEmiRNAs related to the physical activity revealed among 28 enriched GO terms, 10 involved in the transport, localization, and organization of proteins and other cellular components (Fig. 6 and Supplementary table 4). Other pathways were related to mitochondrial organization and protein localization in these organelles, confirming an observed relationship with physical exercise^66^. It is worth pointing out that while most of the studies investigated the effect of physical activity on miRNA expression measured after exercise, our samples were collected randomly during the day. However, our results suggest that also mild differences in physical activity habits may be reflected in different faecal miRNA expression levels, strongly encouraging further analysis on this field with larger cohorts.

Overall, our work highlighted that some of the examined traits differently impact faecal miRNA levels in the two sexes. These differences are mainly observable with BMI and lifestyle habits. We cannot firmly claim that there is an effective inter-sexual difference since we cannot rule out that these results can be ascribed to the different number of individuals in each category, thus influencing the statistics. Nevertheless, these findings suggest the importance of sex as a possible confounder also in miRNA-based studies.

Interestingly, some of the DEmiRNAs overlapped among different comparisons, suggesting that more than one variable can influence their expression in stool samples. The highest number of common DEmiRNAs (n = 20) was observed between BMI and coffee consumption. However, a group of overlapping DEmiRNAs showed opposite trends of expression among different variables, such as those down-regulated with coffee consumption but up-regulated with both increasing BMI and physical activity, and other miRNAs up-regulated in smoking but down-regulated with increasing BMI.

We also assessed individual inter/intra variability of miRNAs detected in stool by deep sequencing. miR-647-3p, miR-6075, and miR-320e-5p showed the lowest variability across the population while nine miRNAs were consistently detected in all samples. Intra-individual analysis of miRNA expression levels showed a good correlation between two samplings performed for six subjects at two time-points, with the second collection performed approximately after one year. Also, the nine miRNAs detected in all samples did not report any significant difference in mean expression levels when compared between the two time-points. Similarly, assessing the overall DEmiRNAs intra-variability in the two time-points, 85.2% of them did not show any significant difference of expression. Our findings suggest that, in healthy conditions, miRNA expression levels detectable in stool seems to be relatively stable over several months. However, within-stool and within-day stool variability are a matter of debate for the validation of biomarkers in this specimen^67^.

The present study relies on a large sample size analysed. The subjects involved were all presumably healthy at recruitment. Moreover, the availability of the data from the questionnaires for all the individuals enabled a consistent statistical analysis with very few missing data. Another strength of this work was the miRNome-wide approach which enabled the analysis of all the currently known and expressed miRNAs: the majority of the previous studies on this field were, in fact, based on arrays or RT-qPCR methods and thus focused only on a limited number of miRNAs.

We are also aware of some limitations: the self-reported information retrieved from the questionnaires may contain data entry errors, or individuals may have wrongly answered. Another limitation is the small sample size for some of the categories investigated among the variables, such as the obese and underweight groups in the BMI analysis, the active category for the physical activity, and non-alcohol consumers. Thus, further investigations in an independent cohort with a similar sample size might be helpful to validate/corroborate our results. However, it is currently difficult to find another cohort with both stool miRNA profile data and detailed information on lifestyle characteristics available.

To conclude, profiles of several miRNAs in stool reflect main common traits and lifestyle habits. These findings are also supported by the pathway enrichment analysis results highlighting the involvement of several DEmiRNAs in biological processes characterising many of the analysed traits. Some of the observed associations confirmed those previously reported in similar studies on other biospecimens while many are hereby observed for the first time, supporting the whole miRNome explorative approach. Currently, we cannot state a relation of causality between the investigated variables and miRNA profiles, as we cannot rule out the potential for other confounding factors. However, considering that a group of DEmiRNAs in our study has already been investigated as potential biomarkers in relation to various diseases and cancers^68, 69^, we may conclude that the role of the described confounders should be carefully considered in studies evaluating the potentiality of faecal miRNAs as molecular biomarkers.

## Methods

### Study population

We collected stool samples from healthy donors participating in different studies running in our laboratory. Briefly, 132 volunteers were recruited from a study investigating the role of different dietary habits described in Tarallo et al.^28^ (Study 1), 76 individuals were recruited as controls in a study on colorectal cancer (i.e., negative at colonoscopy for any colorectal malignancy or polyp, or other gastrointestinal diseases) (Study 2)^70^, and 127 individuals in an association study comparing healthy individuals either on gluten-free diet or with no dietary restrictions (Study 3, not published yet).

All subjects provided written informed consent and filled out, at the time of the recruitment, dietary and lifestyle questionnaires validated in the European Prospective Investigation into Cancer and Nutrition (EPIC) study^71^. These latter were deposited in “*AcQUE*” (https://kdd.di.unito.it/epic), an in-house web-tool specifically developed for the digital acquisition of questionnaire data.

Local Ethics Committees (Azienda Ospedaliera SS. Antonio e Biagio e Cesare Arrigo di Alessandria and Azienda Ospedaliera-Universitaria Città della Salute e della Scienza di Torino, Italy) approved the study.

The present research involving human research participants has been performed in accordance with the Declaration of Helsinki. Human participants' names and other identifiers were removed from all sections of the manuscript, including supplementary information.

### Sample collection

Naturally evacuated stool samples were collected at home from all participants according to the instruction given and using the Norgen Stool Nucleic Acid Collection and Preservation Tubes (Norgen Biotek Corp). Samples were brought to the Italian Institute for Genomic Medicine (IIGM) laboratory or to the recipient hospital. Faecal samples were aliquoted into Eppendorf LoBind tubes and stored at − 80 °C until use.

### Total RNA extraction from stool

Total RNA was extracted from 200 µl faecal aliquots with the Stool total RNA purification kit (Norgen Biotek Corp) using the protocol recommended by the manufacturer. RNA quality and quantity were verified according to the MIQE guidelines (http://miqe.gene-quantification.info/). For all samples, RNA concentration was quantified by Qubit fluorometer with a Qubit microRNA assay kit (Invitrogen).

### Library preparation for small RNA sequencing

Library preparation for sRNA-Seq was performed with the NEBNext Multiplex Small RNA Library Prep Set for Illumina (Protocol E7330, New England BioLabs Inc., USA; New England BioLabs Inc., USA) as described in Tarallo et al.^22^. For each sample, 250 ng of RNA was used as starting material to prepare libraries.

Each library was prepared with a unique indexed primer so that the libraries could all be pooled into one sequencing lane. Multiplex adaptor ligations, reverse transcription primer hybridization, reverse transcription reaction, and PCR amplification were performed according to the protocol for library preparation (Protocol E7330, New England BioLabs Inc., Ipswich, MA, USA). After PCR amplification, the cDNA constructs were purified with the QIAQuick PCR Purification Kit (Qiagen, Germany) following the modifications suggested by the NEBNext Multiplex Small RNA Library Prep Protocol and loaded on the Bioanalyzer 2100 (Agilent Technologies, Milan, Italy) using the DNA High Sensitivity Kit (Agilent, Germany) according to the manufacturer’s protocol. Libraries were pooled together (24-plex) and further purified with a gel size selection.

A final Bioanalyzer 2100 run with the High Sensitivity DNA Kit (Agilent Technologies, Milan, Italy) that allows the analysis of DNA libraries regarding size, purity and concentration completed the workflow of library preparation.

The obtained sequence libraries (24-samples multiplexed) were subjected to the Illumina sequencing pipeline, passing through clonal cluster generation on a single-read flow cell (Illumina Inc., USA) by bridge amplification on the cBot (TruSeq SR Cluster Kit v3-cBOT-HS, Illumina Inc., USA) and 50 cycles sequencing-by-synthesis on the HiSeq 2000 (Illumina Inc., USA) (in collaboration with Genecore Facility at EMBL, Heidelberg, Germany).

### Analysis of miRNAs from sRNA-seq data

A full description of miRNA data analysis is detailed elsewhere^70, 72^. FastQC software was used to check the quality of Fastq files (http://www.bioinformatics.babraham.ac.uk/projects/fastqc/). Reads shorter than 14 nt were discarded. Cutadapt^73^ was used to clip the reads that passed the quality control from the adapter sequences by imposing a maximum error rate in terms of mismatches, insertions, and deletions equal to 0.15. The length of the raw sRNA-Seq reads was 50 bp. Trimmed reads were mapped against an in-house reference of human miRNA sequences composed of 1,917 precursor miRNAs from miRBase v22^74^. The alignment was performed using BWA algorithm v. 0.7.12^75^ with the default settings. Using these settings, the seeding was not performed for reads shorter than 32 bp, and the reads were entirely evaluated for the alignment. miRNAs were annotated and quantified using two methods called the “knowledge-based” and “position-based” methods. In the “knowledge-based” method, the arm starting positions of the miRNA precursors were well defined. Based on the position of the mapped reads, it was possible to quantify the mature miRNAs. Alternatively, the “position-based” method was implemented for those miRNA precursors in which the positions of the 5p and 3p arms are not defined in miRBase. With the second method, based on the position of the read, it was assigned a “-5p” or “-3p” suffix to the name of miRNAs written in Italics (to distinguish from the other miRNAs with assigned arms derived from miRBase). The quantification of mature miRNA annotations was performed by counting the read alignment reported by BWA output and an overall of 3,524 miRNAs were analysed. The results generated by the annotation and quantification methods were merged into a unique mature miRNA count matrix, composed of 3,524 rows (miRNAs) and 335 columns (samples).

### Classification criteria for common traits and lifestyle habits

Information on smoking status, alcohol and coffee consumption, and physical activity were collected from the quantitative and qualitative EPIC dietary and lifestyle questionnaires^71^ whereas individual characteristics (i.e. age, height and weight ) were reported in a baseline questionnaire. For women, menopausal status information (premenopausal and postmenopausal) was also included in EPIC lifestyle questionnaire.

All the investigated traits were treated as categorical variables in the different comparisons, except for age and BMI that were also analysed as continuous variables. According to the distribution of subjects age at recruitment, three age classes were defined based on tertiles. BMI was coded according to the World Health Organization (WHO) guidelines as normal weight (18.5–24.9 kg/m^2^), underweight (< 18.5 kg/m^2^), overweight (25.0–29.9 kg/m^2^), and obese (> 30.0 kg/m^2^) (Table 1).

Self-reported smoking habits were categorized as current, former (all subjects quitted smoking since at least one year), and never smokers. Current smokers were further stratified in light (< 16 cigarettes/day (cigs/day)) and heavy smokers (≥ 16 cigs/day), according to the median number of cigarettes smoked per day (Table 1). Individuals were also categorized by their self-reported alcohol consumption (*i.e.,* gr/day intake of alcohol) in non-drinkers (0 gr/day), low intake (0.1–24.0 gr/day for male and 0.1–12.0 gr/day for female), and high intake (> 24.0 gr/day for male and > 12.0 gr/day for female) according to WHO guidelines. A similar stratification was also adopted according to the coffee consumption: individuals were initially grouped into drinkers and non-drinkers. The former category was then further stratified in low (< 8.0 gr/day) and high (> 8.0 gr/day) intake coffee drinkers according to the median value.

Physical activity was assessed by the total Physical Activity Index (PAI) according to the EPIC guidelines. Briefly, for each subject, the PAI was calculated based on the different sources of recreational, household, and occupational activities self-reported in the questionnaires. The household and recreational activities were combined with metabolic equivalent intensity values (METs)^76^ assigned for each activity, to obtain the MET-hours per week. Total MET-hours per week were divided into sex-specific quartiles (low, medium, high, and very high) and cross-classified with the categories of occupational activity. Finally, each subject was categorized into one of the four following groups: inactive, moderately inactive, moderately active, and active.

### Statistical and computational analyses

All statistical analyses were performed using R software version 4.0.4. The miRNA intra-individual variability was tested by a correlation analysis and a Wilcoxon paired test between miRNA expression levels of a subset of subjects who provided samples at two different time points. To assess the inter-individual variability of miRNA expression levels the mean absolute deviation (MAD) was calculated after filtering out all miRNAs with a median number of normalised reads lower than 1. Then, for each miRNA, a coefficient of variation (CV) was obtained as the ratio between the MAD and the median.

Differential expression analyses were performed with the DESeq2 package (v.1.28.1), using the Wald method as statistical test^77^. Two types of analyses were carried out: the first included all samples, and the second one was performed after stratification by sex. Each model was adjusted for potential confounding biological variables (age and sex in the full model analysis, only age in the stratified analysis) and for any source of possible batch effect (i.e., library pools). A miRNA was defined as significantly differentially expressed if associated with a Benjamini–Hochberg adjusted p-value lower than 0.05 and a median number of normalised reads of at least 10 within at least one of the sample groups considered in the analyses.

Correlations between miRNA (only those with a median number of reads ≥ 10) expression levels and age or BMI, considered as continuous variables, were calculated using the Spearman’s rank correlation coefficient (SCC). Subsequently, miRNAs were classified in three categories, based on their SCC: increasing with age/BMI (SCC > 0.2), unaltered (-0.2 < SCC < 0.2) or decreasing with age/BMI (SCC < -0.2) as reported in^10^.

For DEmiRNAs associated with the analysed variables, the miRNA target functional enrichment analysis was performed with RBiomirGS v0.2.12^78^, using as input the calculated log2 Fold-change (FC) and adjusted p-value from the DE analysis. A detailed description of the analysis is reported elsewhere^79^.

## Supplementary Information

Supplementary Information 1.

Supplementary Information 2.

Supplementary Information 3.

Supplementary Information 4.

## Data Availability

Raw small RNA sequencing data are available upon request to the authors.
